# A Review on Genotoxic and Genoprotective Effects of Biologically Active Compounds of Animal Origin

**DOI:** 10.3390/toxins15020165

**Published:** 2023-02-17

**Authors:** Nikolajs Sjakste, Goran Gajski

**Affiliations:** 1 Department of Medical Biochemistry, Faculty of Medicine, University of Latvia, 1004 Riga, Latvia; 2 Genetics and Bioinformatics, Institute of Biology, University of Latvia, 1004 Riga, Latvia; 3 Mutagenesis Unit, Institute for Medical Research and Occupational Health, 10000 Zagreb, Croatia

**Keywords:** venom, invertebrates, vertebrates, genotoxicity, DNA damage, pharmaceuticals, drugs, therapeutic application

## Abstract

Envenomation by animal venoms remains a serious medical and social problem, especially in tropical countries. On the other hand, animal venoms are widely used as a source of biologically active compounds for the development of novel drugs. Numerous derivatives of animal venoms are already used in clinical practice. When analysing the mechanisms of action of animal venoms, attention is usually focused on the main target of the venom’s enzymes and peptides such as neurotoxic, cytotoxic or haemorrhagic effects. In the present review, we would like to draw attention to the “hidden” effects of animal venoms and their derivatives in regard to DNA damage and/or protection against DNA damage. Alkaloids and terpenoids isolated from sponges such as avarol, ingenamine G or variolin B manifest the capability to bind DNA *in vitro* and produce DNA breaks. Trabectidin, isolated from a sea squirt, also binds and damages DNA. A similar action is possible for peptides isolated from bee and wasp venoms such as mastoparan, melectin and melittin. However, DNA lesions produced by the crude venoms of jellyfish, scorpions, spiders and snakes arise as a consequence of cell membrane damage and the subsequent oxidative stress, whereas certain animal venoms or their components produce a genoprotective effect. Current research data point to the possibility of using animal venoms and their components in the development of various potential therapeutic agents; however, before their possible clinical use the route of injection, molecular target, mechanism of action, exact dosage, possible side effects and other fundamental parameters should be further investigated.

## 1. Introduction

Pharmaceuticals derived from animals continuously make a major contribution to health in terms of prevention and treatment of many diseases [1,2,3,4]. Animal venoms and their components, such as those from snakes, scorpions, spiders, bees, wasps, snails, toads, frogs, lizards and sea anemones have long been used in scientific research and are the basis of many products and drugs that are of great use in medicine today [5,6,7,8,9,10,11,12]. Crude venoms are complex bioactive chemicals rich in proteins and peptides with diverse pharmacological actions that are often protease-resistant due to their disulphide-rich structures. For example, in spider venoms the disulphide bridges form “cysteine knots”, where three bridges stabilize antiparallel beta sheets, drastically increasing the stability of the protein [13]. The components of venoms are specific, stable, potent and have the ability to modify molecular targets, thus making good therapeutic candidates. Animal venoms have been used as a traditional medicine to treat a variety of conditions, including arthritis, rheumatism and chronic pain as well as autoimmune, cardiovascular and skin diseases [3,6,14,15,16,17]. Moreover, one of the most promising fields in venom research from the therapeutic aspect is their use in anticancer research. This is driven by the resistance to chemotherapeutics by cancerous cells that is making cancer treatment more complicated, hence, animal venoms have emerged as an alternative strategy for anticancer therapeutics and could also impact the costs related to cancer treatment [15,18,19,20,21,22,23]. The anticancer activities of animal venoms include the inhibition of the proliferation of cancer cells, their invasion, cell cycle arrest, induction of apoptosis or necrosis and the identification of the involved signalling pathways [14,15,24].

Although there are numerous animal venoms that often show good results towards cancerous cells, there are always open questions regarding their potential toxicity towards normal non-target cells and tissues, making this kind of toxicity one of the greatest obstacles for the possibility of an actual remedy [4,14,25,26,27]. Therefore, the possible genotoxic effects of chemical compounds used as medical remedies are intensively studied. There is a vast body of literature concerning both natural and synthetic compounds [28,29]. Much less attention is paid to the genotoxic effects of animal venoms and other compounds of animal origin, although animal venoms *per se* are in the focus of interest of numerous researchers [4]. However, genotoxic effects can be produced by these compounds due to envenomation or as a side effect of medical remedies. 

**Figure 1:**
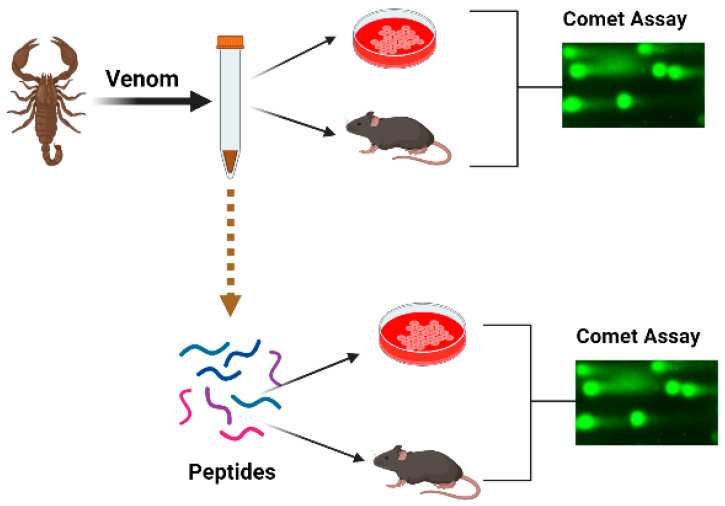
Schematic presentation of the organization of experiments aimed toward the study of the genotoxic effects of venoms.

In this integrative review, we shall try to summarize the accessible data on genotoxic and/or genoprotective activities of the venoms and their components. Data will be reviewed following biological systematics. We conducted a search to identify relevant papers using scientific databases, including PubMed (www.pubmed.com (accessed on 1 February 2023)), Web of Knowledge (www.webofknowledge.com (accessed on 1 February 2023)) and Scopus (www.scopus.com (accessed on 1 February 2023)). The keywords comprised the name of the taxon, the word “venom” and the sought effect, for example “snake venom and DNA damage”, “snake venom and comet assay”. If the individual DNA-damaging components of the venom were revealed, the search was repeated with the name of the compound, for example “trabectidin and DNA damage”, “trabectidin and DNA binding” and “trabectidin and comet assay”, etc. The titles and abstracts were assessed to consider the articles for inclusion in the review, with sixty papers found. We did not apply any restriction concerning the publication language, country or dates of publication. Other relevant original and review papers were also identified from the reference lists of papers found in the search and those papers have been included in the present review and summarized in Table 1. The papers describing comet assay experiments were included if at least 50 cells were analysed per experimental point and several concentrations of the active compound were compared with the negative and positive controls (Figure 1). Papers on the topic first appeared in 2002 and the number of publications has increased since 2010, whereas in the last few years there were four publications yearly. There are one or two publications about one species, with the exception for honeybees where thirteen papers were published. 

## 2. Results of Literature Search

### 2.1. Coelenterata

Venomous jellyfish produce numerous problems for both tourists and fishermen in coastal areas where jellyfish are abundant. The possible genotoxic action of the venom extracted from the Mediterranean jellyfish *Pelagia noctiluca* nematocysts was studied in green monkey kidney (Vero) and human colon cancer (HCT 116) cells at concentrations of 80, 160, 320 and 640 μg/mL. An alkaline comet assay indicated dose-dependent DNA damage. DNA breakage was observed in both cell cultures. It is presumed that the breaks arose due to the oxidative stress triggered by the venom, whereas later DNA lesions favoured the development of apoptosis [53,54]. When non-fractionated nematocysts were incubated with green monkey Vero cells, dose-dependent DNA damage was also observed with a maximum effect produced by 150,000 nematocysts per mL [53].

### 2.2. Sponges (Poryphera)

Sponges possess a very potent secondary metabolism, producing numerous alkaloids, terpenoids and other biologically active compounds. These products compensate for the lack of an immune system in this taxon. Chemical compounds isolated from sponges are tested for various pharmacological effects [55,56]. Although mostly marine sponges are in the focus of interest, freshwater animals also synthesize numerous active compounds. The acetone extract of the freshwater sponge *Ochridaspongia rotunda* manifested cytotoxic and antiradical activities; however, the extract did not induce DNA breaks [57]. Similarly, the extract of the marine sponge *Agelas oroides* produced cytotoxic effects and induced reactive oxygen species (ROS) formation and apoptosis but did not induce DNA breaks [58]. On the contrary, aqueous and alcoholic–aqueous extracts from *Aplysina fulva*, a Brazilian marine sponge, produced DNA breakage in Balb/c 3T3 mouse fibroblasts quantified by the alkaline comet assay compared with untreated cells, whereas the extent of the breakage decreased with the dilution of the extracts (1:2; 1:5; 1:10) [59]. 

The genotoxic effects of individual compounds isolated from sponges also differ from the genotoxicity point of view. The terpenoid avarol from *Dysidea avara* induced DNA breaks in a human cancer (HT29) cell line and Friend leukaemia cells assayed by alkaline single-cell electrophoresis [30,31]. The DNA damaging effect was due to the formation of hydroxyl radicals generated in the quinone/hydroquinone (avarol/avarone) cycle, as the compound does not bind DNA covalently [30]. However, avarol appears to bind DNA non-covalently, both via intercalation and minor groove binding with subsequent DNA damage [60]. 

**Figure 2:**
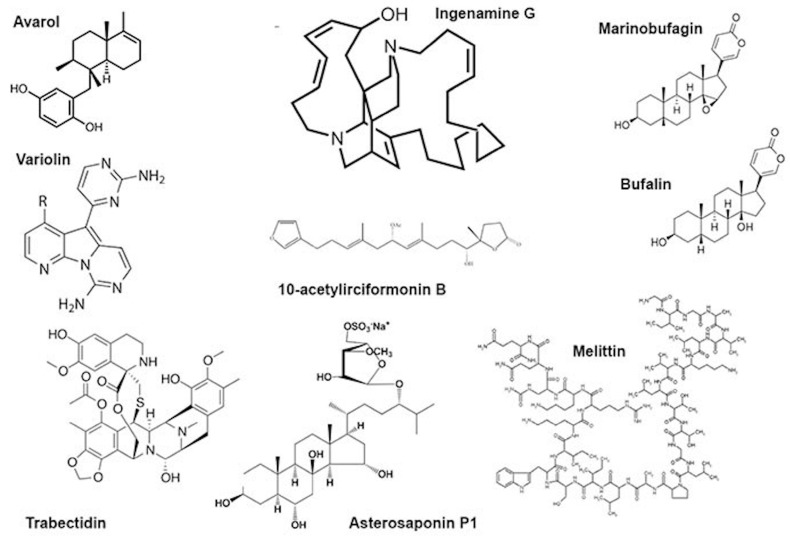
Some low-molecular substances from animal venoms.

Another sponge terpenoid, 10-acetylirciformonin B (Figure 2), produced double-strand breaks detected both by the neutral comet assay and histone γ-H2AX expression in human leukaemia (HL 60) cells; the DNA breakage was followed by apoptosis. In cells treated with the compound at concentrations of 1.25 and 2.5 μg/mL, the mean tail moment in the neutral comet assay increased in a dose-dependent manner compared with the untreated control, and the intensity of the γ-H2AX band followed the same trend [32]. Ingenamine G (Figure 2), an alkaloid isolated from the Brazilian marine sponge *Pachychalina alcaloidifera (Haplosclerida)*, induced DNA breaks in human lymphocytes. The DNA damage was evaluated by alkaline comet assay at concentrations of 5, 10, 15 and 20 μg/mL and compared with the untreated control. It was suggested that the compound affects the construction of a mitotic fuse [33]. According to the results of synchrotron beam diffraction on the crystal variolin B (Figure 2), an alkaloid with anticancer activity from the Antarctic sponge *Kirckpatrickia variolosa* binds DNA via the intercalative mode [34]. Acetylated bile acids from *Siphonochalina fortis* did not induce DNA breaks in human lymphocytes [61]. 

### 2.3. Annelida

Genotoxic effects were produced by tissue extracts of marine polychaetes. DNA breaks were produced in mussel gills by a skin extract from *Hediste diversicolor* and *Glycera alba* jaw proboscis. It was suggested that *H. diversicolor* secretes toxins via the skin for protection against predators, but *G. alba* secretes toxins for predation [62].

### 2.4. Mollusca

The venom of very dangerous snails of the *Conus* genus is composed of hundreds of peptides called conotoxins. Some are used as analgesics, whereas other pharmacological applications are sought [63]. Data on the possible genotoxic action of these species are scarce; however, it was shown that the NMDA receptor agonist ω-conotoxin did not produce DNA damage in the spinal cords and blood cells of Wistar rats. The alkaline comet assay demonstrated the protective effect of the CGX-1007, a recombinant analogue of ω-conotoxin, against DNA damage produced by staurospirine in primary cultures of forebrain neurons [35].

### 2.5. Arachnida

This taxon is represented by numerous venomous animals, scorpions and spiders. Venoms of *Arachnida* are complex mixtures of proteins and low-molecular organic compounds and are mainly neurotoxic [9,64]. 

#### 2.5.1. Scorpions

The venom of a Brazilian scorpion *Tityus stigmurus* produces DNA damage in the blood and testicular cells of Swiss mice, probably by triggering oxidative stress [65], consequently resulting in reproductive disorders in animals and humans. The animals received a single dose of the venom’s 1/2 LD_50_ (0.387 mg/kg), the effects were monitored at different time points up to 48 h, six animals per point. Alkaline comet assays indicated accumulation of DNA breaks, peaking at 2 h for both organs [65]. Similarly, the bolus injection of the venom of the most dangerous Brazilian yellow scorpion (*Tityus serrulatus*) at a dose corresponding to 1/2 LD_50_ (0.90 mg/kg) produced DNA damage in several organs (hippocampus, cortex, striatum, blood, heart, lung, liver and kidney) of Swiss mice. DNA damage was monitored by the alkaline comet assay, the effect appeared one hour after injection and remained at the same level for 12 h, 8–10 animals were taken for a time point [66]. The venom of the Indian black scorpion (*Heterometrus bengalensis* Koch) produced DNA damage in human leukemic cell lines (U937 and K562) leading to apoptosis. Cells were treated with the venom at an IC_50_ concentration (41.5 μg/mL for U937 and 88.3 μg/mL for K562) and the alkaline comet assay was performed 48 h later. Compared with the control cells, the comet tail length increased in the treated U937 cells by 26.4% and to 80.7% in K562 cells [67]. 

#### 2.5.2. Araneae

Despite a great number of venomous spider species, data about the genotoxic effects of these venoms are scarce. There is a report on the genotoxic effects of sphingomyelinase D isolated from venoms of the *Loxosceles* genus spiders on human (HaCaT) keratinocytes. The enzyme binds the plasmatic membrane of the cells and triggers an increase in the intracellular superoxide level, the radical that produces DNA damage [68]. The venom of the *Phoneutria nigriventer* spider manifests analgesic effects. It was shown that the Phα1β peptide isolated from this venom induced DNA damage in the spinal cord cells of Wistar rats but not in white blood cells. The Wistar rats were administered native Phα1β toxin intrathecally at 500 pmol/site and its recombinant analogue at 200, 500 and 1000 pmol/site. Positive control animals received hydrogen peroxide, and negative control animals received phosphate-buffered saline; there were five animals per group. The increased micronucleus frequency in bone marrow cells suggested mutagenic effects [36]. 

### 2.6. Insecta

*Hymenoptera* venoms consist of a complex mixture of chemically or pharmacologically bioactive components including phospholipases, hyaluronidases and mastoparans [69]. Mastoparans, a group of alpha-helical peptides, appear to be the most promising for pharmacological activities [37] since they are able to penetrate into cells and bind DNA [70]. Melectin, an antimicrobial peptide from the venom of the cleptoparasitic bee *Melecta albifrons*, does not exhibit sequence homology with other wasp venom peptides. Melectin manifests antitumour activity, penetrates membranes and binds DNA [38]. The venom of a parasitoid wasp, *Pteromalus puparum,* contains a peptide with endonuclease activity (PpENVP), which inhibits gene expression in transfected cells relying on two activation sites [71].

It is believed that *Hymenoptera* contain substances able to decrease the genotoxic or mutagenic action of other compounds. A protective effect of the Egyptian honeybee (*Apis mellifera lamarckii*) venom against the damaging action of propionic acid (250 mg/kg 3 days) was observed in the neurons of Sprague–Dawley rat pups. DNA damage was assayed by single-cell electrophoresis in alkaline conditions, 0.5 mg/kg of the venom was administered for 4 weeks, and there were 10 pups in a group. Both DNA damage and markers of oxidative stress were decreased [72]. Moreover, the radioprotective effects of honeybee venom (*Apis mellifera*) against 915 MHz microwave-radiation-induced DNA damage in Wistar rat lymphocytes was observed *in vitro* [73]. On the other hand, the *A. mellifera* venom even at low concentrations (0.1, 0.05 and 0.01μg/mL) did not protect HepG2 cells against the genotoxic action of methyl methanesulfonate [74]. In a similar study conducted with venom from the wasp *Polybia paulista*, it was shown that higher concentrations of the venom (10, 5 and 1 μg/mL) were genotoxic *per se*, whereas lower concentrations (1 ng/mL, 100 and 10 pg/mL) were not genotoxic; neither displayed a genoprotective effect. The genotoxic and mutagenic activity of the venom of *P. paulista* could have been caused by phospholipase, mastoparan and hyaluronidase, as these enzymes disrupt the cell membrane and thereby interact with the genetic material of the cells or even facilitate the entrance of other compounds of the venom that can act on the DNA. Furthermore, venom substances are able to trigger inflammatory process and generate ROS that can interact with the DNA [75]. 

Bee venom (*A. mellifera*) and its mayor constituent melittin (Figure 2) are cytotoxic towards human peripheral blood cells and are able to induce morphological changes in the cell membrane, granulation and lysis of the cells. Moreover, they showed increased DNA damage including oxidative DNA damage as well as increased formation of other markers of genomic instability. This genotoxicity coincides with the increased formation of ROS, reduction of glutathione and increased lipid peroxidation as well as phospholipase C activity, indicating the induction of oxidative stress. Melittin itself is also capable of modulating the gene expression patterns of genes involved in the DNA damage response, oxidative stress and apoptosis [25,26,27,39,40,41].

### 2.7. Echinodermata

*Starfish (Asteroidea)* are typical representatives of marine benthic fauna. They are a rich source of various low-molecular weight metabolites, polarsteroids and sphingolipids being the most abundant. It was shown that asterosaponin P1 from *Patiria pectinifera* (4 μM) could significantly increase the DNA damage produced in colorectal carcinoma (HT-29) cells by low-dose X-rays (2 Gy). In the alkaline comet assay, the tail moment increased from 8.6 ± 3.8 (n = 50) in irradiated cells to 29.5 ± 10.6 (n = 50) in the cells additionally treated with asterosaponin P1 [42].

### 2.8. Ascidacea

Trabectidin was identified as an active anticancer compound from the extract of the sea squirt *Ecteinascidia turbinata*. The compound is synthesized by the microbial symbiont of the tunicate. Actually, the compound is widely used as a drug for treatment of malignancies, including soft-tissue sarcomas, ovarian cancer, breast cancer and non-small-cell lung cancer [76]. Trabectedin is a tetrahydroisoquinoline alkaloid that can form a covalent bond with the amino group of a guanine in selected triplets of DNA duplexes and eventually gives rise to double-strand breaks. It binds the minor groove of DNA. Covalent binding of trabectidin triggers a cascade of events that interfere with several transcription factors, DNA binding proteins and DNA repair pathways [77]. The compound induces both transcription- and replication-coupled DNA double-strand breaks (DSBs) detectible by the neutral comet assay as well as the γ-H2AX assay [43]. DNA cleavage is performed by the XPF/ERCC1 nuclease on the strand opposite to that bound by the drug [78]. Trabectidin adducts stabilize double-stranded DNA and stall replication [79]; trabectidin also induces DNA curvatures [80]. 

### 2.9. Pisces

Fish muscles and fat are rich in DNA-protecting compounds. Supplementation of a dog’s diet with fish oil rich in omega-3 fatty acids (1 g per day, 90 days) reduced DNA damage as assayed by alkaline single-cell electrophoresis in peripheral blood lymphocytes from five dogs receiving fish oil compared with four dogs receiving a standard diet. The DNA damage index was reduced already at day 30 and became more pronounced at day 90 [81]. Bioactive oligopeptides (FWKVV and FMPLH) from the protein hydrolysate of the miiuy croaker (*Acanthuriformes*, *Miichthys miiuy*) muscle protected human umbilical vein endothelial cells (HUVECs) against damage produced by hydrogen peroxide. Antioxidant enzymes and molecules were up-regulated, production of radicals was decreased and the DNA damage assayed by alkaline single-cell electrophoresis was attenuated in a dose-dependent manner [44].

### 2.10. Amphibia

The skin secretion of amphibians presents physiologically active molecules to protect them against microorganisms, predators and infections. The Indian common toad (*Bufo melanostictus*, Schneider) skin extract produced significant DNA damage in human myeloid leukemic (U937 and K562) cells, followed by apoptosis. The alkaline comet assay was performed to detect the DNA damage and the extract was administered in a dose corresponding to the IC_50_ [82]. A protein (BMP1) purified from the above species produced DNA damage assayed by single-cell electrophoresis in alkaline conditions and apoptosis in Ehrlich ascites carcinoma (EAC) cells [45]. 

Marinobufagin, a steroid excreted from the skin of toads *Bufo rubescens* and *Bufo marinus,* manifested a selective cytotoxic effect in tumour cell lines; normal cells were much less sensitive to it. However, it did not produce DNA lesions [46]. Similarly, bufalin, a steroid toxin isolated from the Chinese toad (*Bufo gargarizans*), is a topoisomerase II inhibitor; however, it did not damage DNA [47].

### 2.11. Reptiles (Serpentes)

Snake venoms are formed from a complex mixture of enzymes including phospholipases A_2_, metalloproteinases and serine proteases. The venoms also contain three-finger toxins interfering with nerve impulse transduction and some small peptides. The latter component is more abundant in the *Elapidae* family of snakes, the three former components in the *Viperidae* family. Interestingly, the enzyme of the snake venoms produces toxic effects not related to their enzymatic activity [83]. 

Envenomation after Viperid bites is typically followed by strong pain, local swelling and necrosis, blood loss and cardiovascular damage complicated by coagulopathy, and disruption of the blood-clotting system. Viperid venom also causes vascular endothelial damage and haemolysis. Death is caused by a collapse in blood pressure [83].

Genotoxic effects were reported for venoms of several species. The venom of the Iranian viper (*Vipera latifii*) produced DNA damage in human hepatocellular carcinoma (HepG2) cells [84]. The same cell culture was used for testing genotoxic and cytotoxic effects of the toxic component of the Bulgarian sand viper (*Vipera ammodytes meridionalis*) venom—vipoxin. Vipoxin is composed of a basic and toxic phospholipase A_2_ enzyme and an acidic, enzymatically inactive and non-toxic subunit—a vipoxin acidic component. Vipoxin and the vipoxin acidic component produced double-strand DNA breaks, demonstrated by means of the neutral comet assay. The compounds were administered in doses ranging from 0.35 to 2.88 μM and the effect was dose dependent compared with the negative control. The phospholipase A_2_ component was much less genotoxic, although it triggered apoptosis [48]. 

*Bothrops* is a genus of highly venomous pit vipers endemic to Central and South America. Both the crude venoms of these snakes and their components were tested for genotoxic activity. The venom of *Bothrops moojeni*, popularly known as “caiçaca” or “jararacão”, at a concentration close to IC_50_ 4 (μg/mL) produced DNA breaks in Vero cells *in vitro*; the breaks were repaired in 6 h, and the alkaline comet assay was used. However, when injected into Swiss mice, the venom produced non-reparable lesions [85]. *Bothrops brazili*, *Bothrops jararacussu* and *Bothrops atrox* crude venoms also presented genotoxic potential in human lymphocytes, and the latter two increased DNA breakage 5-fold compared with the negative controls in the alkaline comet assay [86]. Individual components of the *Bothrops* venom are also genotoxic. Phospholipase A_2_ bothropstoxin-I from *B. jararacussu* venom produced DNA breaks in human umbilical vein endothelial cells (HUVECs) and prostate-cancer-derived (DU-145) cells (tumour cell line isolated from a metastatic site in the brain). Bothropsin-I was administered in a dose range of 10, 25 and 50 μg/mL, whereas the dose-dependent effect was observed using the alkaline comet assay. The enzyme was also mutagenic and recombinogenic in *Drosophila* [87]. An L-amino acid oxidase from the same venom, BjussuLAAO-II (0.25–5.00 μg/mL), produced DNA breaks in HepG2 and HUVEC cells. The effect was dose dependent in the alkaline comet assay, it was probably produced by triggering oxidative stress [49]. Similar effects for BjussuLAAO-II were also observed in human colorectal adenocarcinoma (Caco-2) cells [50]. Interestingly, the widely used anti-hypertension remedy captopril, a potent angiotensin-converting enzyme inhibitor initially found in *Bothrops jararaca* venom, produced both double-strand breaks and single-strand DNA breaks in human lymphocytes and macrophages. The level of the double-strand breaks was evaluated by means of the fluorescence assay, whereas single-strand DNA breaks were evaluated by the alkaline comet assay [51].

Bushmaster venom (*Crotalidae*, *Lachesis* genus) is also genotoxic. The venom of the largest venomous snake in South America, *Lachesis muta muta,* produces DNA damage in human lymphocytes [88]. The venom of the rattle snake (*Crotalus durissus terrificus*) belonging to the same subfamily is also genotoxic. Isolated toxins were even more genotoxic than the crude venom, the main toxin of crotalus venom, crotoxin, is composed of an acidic, non-toxic and non-enzymatic subunit (CA) and a basic, weakly toxic, phospholipase A_2_ protein (CB). Crotoxin and its subunits produced DNA breaks in human lymphocytes detected by the alkaline comet assay. Crotamine, another Crotalus toxin, is a 42-residue long protein containing 11 basic residues (9 lysines, 2 arginines) and 6 cysteines that acts on the cell membrane’s sodium channels, is slightly analgesic and is myotoxic, i.e., it penetrates the cells of muscles and promotes necrosis. The alkaline comet assay revealed that the toxin produced DNA breaks in human lymphocytes [52].

The venom of *Philodryas* patagoniensis, a much less dangerous snake from the *Colubridae* family, also produces genotoxic effects. It appears that the cysteine-rich secretory protein (CRISPs) is the main toxin of the venom, inhibiting smooth muscle contraction and cyclic nucleotide-gated ion channels [89]. The alkaline comet assay revealed DNA breaks produced by the venom in human mononuclear cells, probably by triggering oxidative stress [90]. 

Unfortunately, we could not find any data about the genotoxicity of the neurotoxic *Elapidae* family venoms. Concerning the haemolytic venoms reported above, it seems that their genotoxic action is indirect: it is produced via cell damage and production of oxidative stress. Interestingly, antioxidants such as ascorbic [88], caffeic and syringic acids [91] decrease the damaging effects of the snake toxins. 

## 3. Conclusions and Future Perspectives 

**Figure 3:**
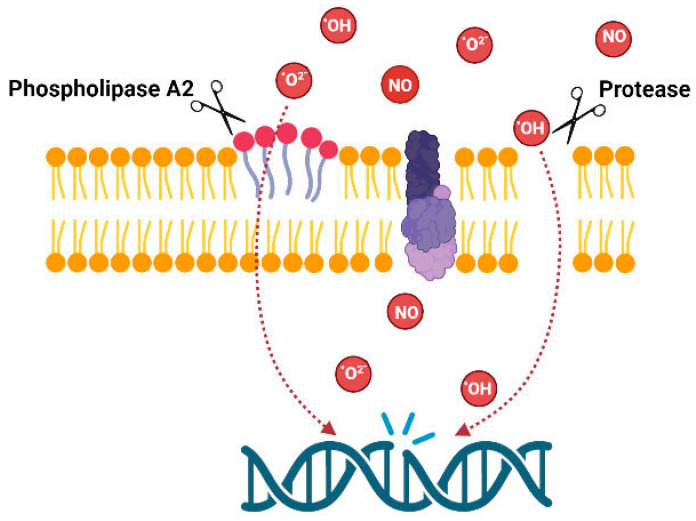
Possible mechanism of genotoxic action of the venom enzymes.

The overview of the above data indicates that data are fragmentary; venoms of animals from several taxons were not tested for genotoxicity. There are no data about fish venoms, molluscan venoms or venoms of myriapods, etc. Data on snakes and amphibians are also fragmentary. However, the presented data clearly indicate the importance of such studies. From the point of view of the mechanisms of action, only some compounds isolated from marine invertebrates such as avarol and trabectidin are DNA binders, thus DNA damage is produced by the compounds themselves. Venoms of other animals act indirectly by producing membrane damage or inducing oxidative stress; consequently, DNA damage arises as a secondary effect (Figure 3). 

Due to venoms’ non-specific toxicity, their therapeutic potential cannot be achieved without a proper delivery vehicle. This could be overcome by nanoparticles that possess the ability to safely deliver a significant amount of venom and/or their components intravenously to target and kill tumour cells [92,93]. Another possibility is a combination drug therapy using the existing chemotherapeutic agents with venom components, which could be useful from the aspect of minimizing concentrations of standard chemotherapeutic drugs during chemotherapy [18,19,94,95]. Since it can be suggested that the future prospects of cancer treatment could lie in combination therapy, it should also be noted that such combinations might lead to the development of toxicities, which need to be evaluated along with the observed anticancer or other therapeutic potentials. Current research data point to the possibility of using animal venoms in the development of antitumour drugs as well as other potential therapeutic agents; several FDA-approved drugs derived from venom peptides or proteins already exist [3]. However, before its possible clinical use, the route of injection, molecular targets, mechanisms of action, exact dosage, possible side effects and other fundamental parameters should be further investigated. Moreover, making these molecules applicable requires extensive preclinical trials, with some applications also demanding clinical trials [96]. 

Moreover, several new techniques, including bioinformatics tools and *in silico* analysis, can support the development of new therapeutic agents based on animal biodiversity aimed at large-scale prediction of erythrocyte lysis induced by peptides. Hence, many online databases filled with peptide sequences and their biological metadata have paved the way toward haemolysis prediction using user-friendly, fast-access machine-learning-driven programs. Although the development of such predictive approaches to peptide toxicity has only just started, their contributions demonstrate the large potential for peptide science and computer-aided drug design in the identification of selective, non-toxic peptide therapeutics. Nevertheless, these new approaches must consider predicting the balance between toxicity and therapeutic effect. Hence, the future design of peptide pharmaceuticals should include the interplay between computational, *in vitro* and *in vivo* approaches [97,98,99,100].

Due to the enormous venom diversity, further research is needed, and our knowledge in this regard is still limited. For the application of biologically active compounds of animal origin as pharmacological tools and medications, the development of innovative approaches and best practices for target identification will be critical.

## Figures and Tables

**Table 1:** Genotoxic and genoprotective effects of animal venom components with pharmacological action.

Compound	Source	Genotoxic/Genoprotective Effect	Cell Types	Pharmacological Action	References
Avarol	*Dysidea avara*	Genotoxic	Human cancer (HT29) cell line and Friend leukaemia cells	Antitumour, antibacterial, antiviral	[30,31]
10-Acetylirciformonin B	*Ircinia* sp.	Genotoxic	Human cancer (HT29) cell line	Antitumour	[32]
Ingenamine G	*Pachychalina alcaloidifera*	Genotoxic	Human lymphocytes	Antitumour	[33]
Variolin B	*Kirckpatrickia variolosa*	Genotoxic	Human cancer cells	Antitumour	[34]
ω-Conotoxin	*Conus magus*	Genoprotective	Primary cultures of rat forebrain neurons	Analgesic	[35]
Phα1β peptide	*Phoneutria nigriventer*	Genotoxic	Rat spinal cord cells	Analgesic	[36]
Mastoparans	*Polibia paulista*, *Vespa* sp.	Genotoxic	Cancer cell lines	Antitumour, antimicrobial, antiviral	[37]
Melectin	*Melecta albifrons*	Genotoxic	Cancer cell lines	Antimicrobial, antitumour	[38]
Melittin	*Apis mellifera*	Genotoxic	Human peripheral blood cells	Antitumour	[25,26,27,39,40,41]
Asterosaponin P1	*Patiria pectinifera*	Genotoxic	Colorectal carcinoma cells HT-29	Radiomimetic	[42]
Trabectidin	*Ecteinascidia turbinata*	Genotoxic	Human cancer cells	Antitumour	[43]
FWKVV and FMPLH	*Miichthys miiuy*	Genoprotectiuve	HUVEC cells	Antimutagenic	[44]
BMP1	*Bufo melanostictus*	Genotoxic	Ehrlich ascites cells	Antitumour	[45]
Marinobufagin	*Bufo rubescens* *Bufo marinus*	Non-genotoxic	Human cancer cells	Antitumour	[46]
Bufalin	*Bufo gargarizans*	Non-genotoxic	Human cancer cells	Antitumour	[47]
Vipoxin	*Vipera ammodytes meridionalis*	Genotoxic	Human hepatocellular carcinoma (HepG2)	Neurotoxic	[48]
BjussuLAAO-II	*Bothrops jararacussu*	Genotoxic	HepG2 HUVEC Caco-2 cells	Antitumour	[49,50]
Captopril	*Bothrops jararaca*	Genotoxic	Human lymphocytes Human macrophages	Antihypertensive	[51]
Crotoxin	*Crotalus durissus terrificus*	Genotoxic	Human lymphocytes	Immunomodulatory, anti-inflammatory, anti-microbial, antitumour and analgesic	[52]

## Data Availability

Not applicable.

## References

[1] McDermott A. (2020). News Feature: Venom Back in Vogue as a Wellspring for Drug Candidates. Proc. Natl. Acad. Sci. USA.

[2] Holford M., Daly M., King G.F., Norton R.S. (2018). Venoms to the Rescue. Science.

[3] King G.F. (2011). Venoms as a Platform for Human Drugs: Translating Toxins into Therapeutics. Expert Opin. Biol. Ther..

[4] von Reumont B.M., Anderluh G., Antunes A., Ayvazyan N., Beis D., Caliskan F., Crnković A., Damm M., Dutertre S., Ellgaard L. (2022). Modern Venomics-Current Insights, Novel Methods, and Future Perspectives in Biological and Applied Animal Venom Research. Gigascience.

[5] Abd El-Aziz T.M., Garcia Soares A., Stockand J.D. (2019). Snake Venoms in Drug Discovery: Valuable Therapeutic Tools for Life Saving. Toxins.

[6] Son D.J., Lee J.W., Lee Y.H., Song H.S., Lee C.K., Hong J.T. (2007). Therapeutic Application of Anti-Arthritis, Pain-Releasing, and Anti-Cancer Effects of Bee Venom and Its Constituent Compounds. Pharmacol. Ther..

[7] Kalita B., Saviola A.J., Mukherjee A.K. (2021). From Venom to Drugs: A Review and Critical Analysis of Indian Snake Venom Toxins Envisaged as Anticancer Drug Prototypes. Drug Discov. Today.

[8] Qi J., Zulfiker A.H.M., Li C., Good D., Wei M.Q. (2018). The Development of Toad Toxins as Potential Therapeutic Agents. Toxins.

[9] Saez N.J., Senff S., Jensen J.E., Er S.Y., Herzig V., Rash L.D., King G.F. (2010). Spider-Venom Peptides as Therapeutics. Toxins.

[10] Muttenthaler M., King G.F., Adams D.J., Alewood P.F. (2021). Trends in Peptide Drug Discovery. Nat. Rev. Drug Discov..

[11] Harvey A. (1998). From Demons to Darlings: Drugs from Venoms. Drug Discov. Today.

[12] Gajski G., Čimbora-Zovko T., Osmak M., Garaj-Vrhovac V., Viktorsson K. (2012). Bee Venom and Melittin Are Cytotoxic against Different Types of Tumor and Non-Tumor Cell Lines In Vitro. Advancements in Cancer Research.

[13] Herzig V., King G.F. (2015). The Cystine Knot Is Responsible for the Exceptional Stability of the Insecticidal Spider Toxin ω-Hexatoxin-Hv1a. Toxins.

[14] Gajski G., Garaj-Vrhovac V. (2013). Melittin: A Lytic Peptide with Anticancer Properties. Environ. Toxicol. Pharmacol..

[15] Chatterjee B. (2018). Animal Venoms Have Potential to Treat Cancer. Curr. Top. Med. Chem..

[16] Lewis R.J., Garcia M.L. (2003). Therapeutic Potential of Venom Peptides. Nat. Rev. Drug Discov..

[17] Greener M. (2020). The next Generation of Venom-based Drugs. Prescriber.

[18] Gajski G., Čimbora-Zovko T., Rak S., Rožman M., Osmak M., Garaj-Vrhovac V. (2014). Combined Antitumor Effects of Bee Venom and Cisplatin on Human Cervical and Laryngeal Carcinoma Cells and Their Drug Resistant Sublines. J. Appl. Toxicol..

[19] Gajski G., Čimbora-Zovko T., Rak S., Osmak M., Garaj-Vrhovac V. (2016). Antitumour Action on Human Glioblastoma A1235 Cells through Cooperation of Bee Venom and Cisplatin. Cytotechnology.

[20] Roy A., Bharadvaja N. (2021). Venom-Derived Bioactive Compounds as Potential Anticancer Agents: A Review. Int. J. Pept. Res. Ther..

[21] Chaisakul J., Hodgson W.C., Kuruppu S., Prasongsook N. (2016). Effects of Animal Venoms and Toxins on Hallmarks of Cancer. J. Cancer.

[22] Viegas S., Ladeira C., Costa-Veiga A., Perelman J., Gajski G. (2017). Forgotten Public Health Impacts of Cancer—An Overview. Arch. Ind. Hyg. Toxicol..

[23] Norouzi P., Mirmohammadi M., Houshdar Tehrani M.H. (2022). Anticancer Peptides Mechanisms, Simple and Complex. Chem. Biol. Interact..

[24] Oršolić N. (2012). Bee Venom in Cancer Therapy. Cancer Metastasis Rev..

[25] Gajski G., Domijan A.-M., Žegura B., Štern A., Gerić M., Novak Jovanović I., Vrhovac I., Madunić J., Breljak D., Filipič M. (2016). Melittin Induced Cytogenetic Damage, Oxidative Stress and Changes in Gene Expression in Human Peripheral Blood Lymphocytes. Toxicon.

[26] Gajski G., Garaj-Vrhovac V. (2011). Bee Venom Induced Cytogenetic Damage and Decreased Cell Viability in Human White Blood Cells after Treatment in Vitro: A Multi-Biomarker Approach. Environ. Toxicol. Pharmacol..

[27] Garaj-Vrhovac V., Gajski G. (2009). Evaluation of the Cytogenetic Status of Human Lymphocytes after Exposure to a High Concentration of Bee Venom in Vitro. Arch. Hig. Rada Toksikol..

[28] Sjakste N., Djelić N., Dzintare M., Živković L. (2020). DNA-BINDING and DNA-Protecting Activities of Small Natural Organic Molecules and Food Extracts. Chem. Biol. Interact..

[29] Muhamedejevs R., Živković L., Dzintare M., Sjakste N. (2021). DNA-Binding Activities of Compounds Acting as Enzyme Inhibitors, Ion Channel Blockers and Receptor Binders. Chem. Biol. Interact..

[30] Sladic D., Gasic M. (2006). Reactivity and Biological Activity of the Marine Sesquiterpene Hydroquinone Avarol and Related Compounds from Sponges of the Order Dictyoceratida. Molecules.

[31] Pejin B., Iodice C., Kojic V., Jakimov D., Lazovic M., Tommonaro G. (2016). In Vitro Evaluation of Cytotoxic and Mutagenic Activity of Avarol. Nat. Prod. Res..

[32] Su J.-H., Chang W.-B., Chen H.-M., El-Shazly M., Du Y.-C., Kung T.-H., Chen Y.-C., Sung P.-J., Ho Y.-S., Kuo F.-W. (2012). 10-Acetylirciformonin B, A Sponge Furanoterpenoid, Induces DNA Damage and Apoptosis in Leukemia Cells. Molecules.

[33] Cavalcanti B.C., Sombra C.M.L., de Oliveira J.H.H.L., de Berlinck R.G.S., de Moraes M.O., Pessoa C. (2008). Cytotoxicity and Genotoxicity of Ingenamine G Isolated from the Brazilian Marine Sponge Pachychalina Alcaloidifera. Comp. Biochem. Physiol. Part C: Toxicol. Pharmacol..

[34] Canals A., Arribas-Bosacoma R., Albericio F., Álvarez M., Aymamí J., Coll M. (2017). Intercalative DNA Binding of the Marine Anticancer Drug Variolin B. Sci. Rep..

[35] Dave J.R., Williams A.J., Moffett J.R., Koenig M.L., Tortella F.C. (2003). Studies on Neuronal Apoptosis in Primary Forebrain Cultures: Neuroprotective/Anti-Apoptotic Action of NR2B NMDA Antagonists. Neurotox. Res..

[36] de Souza A.H., da Rosa L.G., Uliano M.R., da Silva Prado L., Ferraz A.G., Conter L.U., Grivicich I., Dallegrave E., Gomez M.V., Picada J.N. (2019). Evaluation of DNA Damage in Spinal Cord and Mutagenic Effect of a Phα1β Recombinant Toxin with Analgesic Properties from the Phoneutria Nigriventer Spider. Basic Clin. Pharmacol. Toxicol..

[37] de Santana C.J.C., Pires Júnior O.R., Fontes W., Palma M.S., Castro M.S. (2022). Mastoparans: A Group of Multifunctional α-Helical Peptides With Promising Therapeutic Properties. Front. Mol. Biosci..

[38] Liang X., Yan J., Lu Y., Liu S., Chai X. (2021). The Antimicrobial Peptide Melectin Shows Both Antimicrobial and Antitumor Activity via Membrane Interference and DNA Binding. Drug Des. Devel. Ther..

[39] Gajski G., Domijan A.-M., Garaj-Vrhovac V. (2012). Alterations of GSH and MDA Levels and Their Association with Bee Venom-Induced DNA Damage in Human Peripheral Blood Leukocytes. Environ. Mol. Mutagen..

[40] Gajski G., Garaj-Vrhovac V. (2008). Genotoxic Potential of Bee Venom (Apis Mellifera) on Human Peripheral Blood Lymphocytes in Vitro Using Single Cell Gel Electrophoresis Assay. J. Environ. Sci. Health A Tox Hazard Subst. Environ. Eng..

[41] Gajski G., Garaj-Vrhovac V. (2010). Increased Frequency of Sister Chromatid Exchanges and Decrease in Cell Viability and Proliferation Kinetics in Human Peripheral Blood Lymphocytes after In Vitro Exposure to Whole Bee Venom. J. Environ. Sci. Health A Tox Hazard Subst. Environ. Eng..

[42] Malyarenko O.S., Malyarenko T.V., Kicha A.A., Ivanchina N.V., Ermakova S.P. (2019). Effects of Polar Steroids from the Starfish Patiria (=Asterina) Pectinifera in Combination with X-Ray Radiation on Colony Formation and Apoptosis Induction of Human Colorectal Carcinoma Cells. Molecules.

[43] Guirouilh-Barbat J., Redon C., Pommier Y. (2008). Transcription-Coupled DNA Double-Strand Breaks Are Mediated via the Nucleotide Excision Repair and the Mre11-Rad50-Nbs1 Complex. Mol. Biol. Cell.

[44] Wang Y.-Z., Wang Y.-M., Pan X., Chi C.-F., Wang B. (2021). Antioxidant Mechanisms of the Oligopeptides (FWKVV and FMPLH) from Muscle Hydrolysate of Miiuy Croaker against Oxidative Damage of HUVECs. Oxid. Med. Cell. Longev..

[45] Bhattacharjee P., Giri B., Gomes A. (2011). Apoptogenic Activity and Toxicity Studies of a Cytotoxic Protein (BMP1) from the Aqueous Extract of Common Indian Toad (Bufo Melanostictus Schneider) Skin. Toxicon.

[46] da Machado K.C., de Sousa L.Q., Lima D.J.B., Soares B.M., Cavalcanti B.C., Maranhão S.S., Noronha J., de Jesus Rodrigues D., Militão G.C.G., Chaves M.H. (2018). Marinobufagin, a Molecule from Poisonous Frogs, Causes Biochemical, Morphological and Cell Cycle Changes in Human Neoplasms and Vegetal Cells. Toxicol. Lett..

[47] Pastor N., Domínguez I., Mateos S., Cortés F. (2002). A Comparative Study of Genotoxic Effects of Anti-Topoisomerase II Drugs ICRF-193 and Bufalin in Chinese Hamster Ovary Cells. Mutat. Res..

[48] Doumanov J., Mladenova K., Topouzova-Hristova T., Stoitsova S., Petrova S. (2015). Effects of Vipoxin and Its Components on HepG2 Cells. Toxicon.

[49] Machado A.R.T., Aissa A.F., Ribeiro D.L., Costa T.R., Ferreira Jr. R.S., Sampaio S.V., Antunes L.M.G. (2019). Cytotoxic, Genotoxic, and Oxidative Stress-Inducing Effect of an l-Amino Acid Oxidase Isolated from Bothrops Jararacussu Venom in a Co-Culture Model of HepG2 and HUVEC Cells. Int. J. Biol. Macromol..

[50] Machado A.R.T., Aissa A.F., Ribeiro D.L., Hernandes L.C., Machado C.S., Bianchi M.L.P., Sampaio S.V., Antunes L.M.G. (2018). The Toxin BjussuLAAO-II Induces Oxidative Stress and DNA Damage, Upregulates the Inflammatory Cytokine Genes TNF and IL6, and Downregulates the Apoptotic-Related Genes BAX, BCL2 and RELA in Human Caco-2 Cells. Int. J. Biol. Macromol..

[51] de Moura Leão M.F., Duarte J.A., Sauzen P.D., da Piccoli J.C.E., de Oliveira L.F.S., Machado M.M. (2018). Cytotoxic and Genotoxic Effects of Antihypertensives Distributed in Brazil by Social Programs: Are They Safe? Environ. Toxicol. Pharmacol..

[52] Marcussi S., Santos P.R.S., Menaldo D.L., Silveira L.B., Santos-Filho N.A., Mazzi M.V., da Silva S.L., Stábeli R.G., Antunes L.M.G., Soares A.M. (2011). Evaluation of the Genotoxicity of Crotalus Durissus Terrificus Snake Venom and Its Isolated Toxins on Human Lymphocytes. Mutat. Res..

[53] Ayed Y., Boussabbeh M., Zakhama W., Bouaziz C., Abid S., Bacha H. (2011). Induction of Cytotoxicity of Pelagia Noctiluca Venom Causes Reactive Oxygen Species Generation, Lipid Peroxydation Induction and DNA Damage in Human Colon Cancer Cells. Lipids Health Dis..

[54] Ayed Y., Bouaziz C., Brahmi D., Zaid C., Abid S., Bacha H. (2014). Cell Death in Relation to DNA Damage after Exposure to the Jellyfish Pelagia Noctiluca Nematocysts. Environ. Toxicol..

[55] Anjum K., Abbas S.Q., Shah S.A.A., Akhter N., Batool S., Hassan S.S.U. (2016). Marine Sponges as a Drug Treasure. Biomol. Ther..

[56] Varijakzhan D., Loh J.-Y., Yap W.-S., Yusoff K., Seboussi R., Lim S.-H.E., Lai K.-S., Chong C.-M. (2021). Bioactive Compounds from Marine Sponges: Fundamentals and Applications. Mar. Drugs.

[57] Talevska A., Pejin B., Kojic V., Beric T., Stankovic S. (2018). A Contribution to Pharmaceutical Biology of Freshwater Sponges. Nat. Prod. Res..

[58] Ferretti C., Marengo B., De Ciucis C., Nitti M., Pronzato M., Marinari U., Pronzato R., Manconi R., Domenicotti C. (2007). Effects of Agelas Oroides and Petrosia Ficiformis Crude Extracts on Human Neuroblastoma Cell Survival. Int. J. Oncol..

[59] Aiub C., Giannerini A., Ferreira F., Mazzei J., Stankevicins L., Lobo-Hajdu G., Guimarães P., Hajdu E., Felzenszwalb I. (2006). Genotoxic Evaluation of Extracts from Aplysina Fulva, a Brazilian Marine Sponge. Mutat. Res..

[60] Vujčić M.T., Tufegdžić S., Novaković I., Djikanović D., Gašić M.J., Sladić D. (2013). Studies on the Interactions of Bioactive Quinone Avarone and Its Methylamino Derivatives with Calf Thymus DNA. Int. J. Biol. Macromol..

[61] Patiño Cano L.P., Bartolotta S.A., Casanova N.A., Siless G.E., Portmann E., Schejter L., Palermo J.A., Carballo M.A. (2013). Isolation of Acetylated Bile Acids from the Sponge Siphonochalina Fortis and DNA Damage Evaluation by the Comet Assay. Steroids.

[62] D’Ambrosio M., Ramos Í., Martins C., Costa P.M. (2022). An Investigation into the Toxicity of Tissue Extracts from Two Distinct Marine Polychaeta. Toxicon X.

[63] Jin A.-H., Muttenthaler M., Dutertre S., Himaya S.W.A., Kaas Q., Craik D.J., Lewis R.J., Alewood P.F. (2019). Conotoxins: Chemistry and Biology. Chem. Rev..

[64] Escoubas P., Diochot S., Corzo G. (2000). Structure and Pharmacology of Spider Venom Neurotoxins. Biochimie.

[65] Silva M.A., Souza T.G., Melo M.E.G., Silva J.M., Lima J.R., Lira A.F.A., de Aguiar-Júnior F.C.A., Martins R.D., Jorge R.J.B., Chagas C.A. (2020). Tityus Stigmurus Venom Causes Genetic Damage in Blood and Testicular Cells and Affects the Number and Morphology of Gametogenic Lineage Cells in Mice. Toxicon.

[66] Galvani N.C., Vilela T.C., Domingos A.C., Fagundes M.Í., Bosa L.M., Della Vechia I.C., Scussel R., Pereira M., Steiner B.T., Damiani A.P. (2017). Genotoxicity Evaluation Induced by Tityus Serrulatus Scorpion Venom in Mice. Toxicon.

[67] Das Gupta S., Debnath A., Saha A., Giri B., Tripathi G., Vedasiromoni J.R., Gomes A., Gomes A. (2007). Indian Black Scorpion (Heterometrus Bengalensis Koch) Venom Induced Antiproliferative and Apoptogenic Activity against Human Leukemic Cell Lines U937 and K562. Leuk. Res..

[68] da Silva M.S., Lopes P.H., Elias M.C., Tambourgi D. (2020). V Cytotoxic and Genotoxic Effects on Human Keratinocytes Triggered by Sphingomyelinase D from Loxosceles Venom. Arch. Toxicol..

[69] Luo L., Kamau P.M., Lai R. (2022). Bioactive Peptides and Proteins from Wasp Venoms. Biomolecules.

[70] Niidome T., Urakawa M., Takaji K., Matsuo Y., Ohmori N., Wada A., Hirayama T., Aoyagi H. (1999). Influence of Lipophilic Groups in Cationic Alpha-Helical Peptides on Their Abilities to Bind with DNA and Deliver Genes into Cells. J. Pept. Res..

[71] Wang J., Yan Z., Xiao S., Wang B., Fang Q., Schlenke T., Ye G. (2021). Characterization of a Cell Death-Inducing Endonuclease-like Venom Protein from the Parasitoid Wasp Pteromalus Puparum (Hymenoptera: Pteromalidae). Pest Manag. Sci..

[72] Khalil S.R., Abd-Elhakim Y.M., Selim M.E., Al-Ayadhi L.Y. (2015). Apitoxin Protects Rat Pups Brain from Propionic Acid-Induced Oxidative Stress: The Expression Pattern of Bcl-2 and Caspase-3 Apoptotic Genes. Neurotoxicology.

[73] Gajski G., Garaj-Vrhovac V. (2009). Radioprotective Effects of Honeybee Venom (Apis Mellifera) Against 915-MHz Microwave Radiation-Induced DNA Damage in Wistar Rat Lymphocytes: In Vitro Study. Int. J. Toxicol..

[74] Hoshina M.M., Marin-Morales M.A. (2014). Anti-Genotoxicity and Anti-Mutagenicity of Apis Mellifera Venom. Mutat. Res. Genet. Toxicol. Environ. Mutagen..

[75] Hoshina M.M., Santos L.D., Palma M.S., Marin-Morales M.A. (2013). Cytotoxic, Genotoxic/Antigenotoxic and Mutagenic/Antimutagenic Effects of the Venom of the Wasp Polybia Paulista. Toxicon.

[76] Wang J., Wang P., Zeng Z., Lin C., Lin Y., Cao D., Ma W., Xu W., Xiang Q., Luo L. (2022). Trabectedin in Cancers: Mechanisms and Clinical Applications. Curr. Pharm. Des..

[77] D’Incalci M., Galmarini C.M. (2010). A Review of Trabectedin (ET-743): A Unique Mechanism of Action. Mol. Cancer Ther..

[78] Feuerhahn S., Giraudon C., Martínez-Díez M., Bueren-Calabuig J.A., Galmarini C.M., Gago F., Egly J.-M. (2011). XPF-Dependent DNA Breaks and RNA Polymerase II Arrest Induced by Antitumor DNA Interstrand Crosslinking-Mimetic Alkaloids. Chem. Biol..

[79] Bueren-Calabuig J.A., Giraudon C., Galmarini C.M., Egly J.M., Gago F. (2011). Temperature-Induced Melting of Double-Stranded DNA in the Absence and Presence of Covalently Bonded Antitumour Drugs: Insight from Molecular Dynamics Simulations. Nucleic Acids Res..

[80] Mills A., Gago F. (2021). Insight into the Sequence-Specific Elements Leading to Increased DNA Bending and Ligase-Mediated Circularization Propensity by Antitumor Trabectedin. J. Comput. Aided. Mol. Des..

[81] Pellegrino F.J., Risso A., Corrada Y., Gambaro R.C., Seoane A.I. (2021). Influence of Dietary Fish Oil Supplementation on DNA Damage in Peripheral Blood Lymphocytes of Nine Healthy Dogs. Vet. Rec. Open.

[82] Giri B., Gomes A., Debnath A., Saha A., Biswas A.K., Dasgupta S.C., Gomes A. (2006). Antiproliferative, Cytotoxic and Apoptogenic Activity of Indian Toad (Bufo Melanostictus, Schneider) Skin Extract on U937 and K562 Cells. Toxicon.

[83] Ferraz C.R., Arrahman A., Xie C., Casewell N.R., Lewis R.J., Kool J., Cardoso F.C. (2019). Multifunctional Toxins in Snake Venoms and Therapeutic Implications: From Pain to Hemorrhage and Necrosis. Front. Ecol. Evol..

[84] Moridikia A., Zargan J., Sobati H., Goodarzi H.R., Hajinourmohamadi A. (2018). Anticancer and Antibacterial Effects of Iranian Viper (Vipera Latifii) Venom; an In-Vitro Study. J. Cell. Physiol..

[85] Novak Zobiole N., Caon T., Wildgrube Bertol J., de Pereira C.A.S., Okubo B.M., Moreno S.E., Tramontini Gomes de Sousa Cardozo F. (2015). In Vitro and in Vivo Genotoxic Evaluation of Bothrops Moojeni Snake Venom. Pharm. Biol..

[86] Marcussi S., Stábeli R.G., Santos-Filho N.A., Menaldo D.L., Silva Pereira L.L., Zuliani J.P., Calderon L.A., da Silva S.L., Antunes L.M.G., Soares A.M. (2013). Genotoxic Effect of Bothrops Snake Venoms and Isolated Toxins on Human Lymphocyte DNA. Toxicon.

[87] Naves M.P.C., de Morais C.R., de Freitas V., Ribeiro D.L., Lopes D.S., Antunes L.M.G., de Melo Rodrigues V., de Rezende A.A.A., Spanó M.A. (2021). Mutagenic and Genotoxic Activities of Phospholipase A2 Bothropstoxin-I from Bothrops Jararacussu in Drosophila Melanogaster and Human Cell Lines. Int. J. Biol. Macromol..

[88] Cardoso Trento M.V., de Faria Eleutério M.W., Silva Abreu T., Andrade Machado G.H., Cesar P.H.S., Assaid Simão A., Marcussi S. (2019). The Protective Effect Exerted by Ascorbic Acid on DNA Fragmentation of Human Leukocytes Induced by Lachesis Muta Muta Venom. J. Cell Biochem.

[89] Modahl C.M., Saviola A.J., Mackessy S.P. (2021). Integration of Transcriptomic and Proteomic Approaches for Snake Venom Profiling. Expert Rev. Proteom..

[90] Costa M.T., da Silva Goulart A., Salgueiro A.C.F., da Rosa H.S., Perazzo G.X., Folmer V. (2022). Cytotoxicity and Inflammation Induced by Philodryas Patagoniensis Venom. Comp. Biochem. Physiol. C Toxicol. Pharmacol..

[91] Abreu T.S., Braga M.A., Simão A.A., Trento M.V.C., de Eleutério M.W.F., Silva Pereira L.L., Cunha E.F.F., Marcussi S. (2020). Mitochondriotropic Action and DNA Protection: Interactions between Phenolic Acids and Enzymes. J. Biochem. Mol. Toxicol..

[92] Pan H., Soman N.R., Schlesinger P.H., Lanza G.M., Wickline S.A. (2011). Cytolytic Peptide Nanoparticles (‘NanoBees’) for Cancer Therapy. WIREs Nanomed. Nanobiotechnol..

[93] Dabbagh Moghaddam F., Akbarzadeh I., Marzbankia E., Farid M., Khaledi L., Reihani A.H., Javidfar M., Mortazavi P. (2021). Delivery of Melittin-Loaded Niosomes for Breast Cancer Treatment: An in Vitro and in Vivo Evaluation of Anti-Cancer Effect. Cancer Nanotechnol..

[94] Duffy C., Sorolla A., Wang E., Golden E., Woodward E., Davern K., Ho D., Johnstone E., Pfleger K., Redfern A. (2020). Honeybee Venom and Melittin Suppress Growth Factor Receptor Activation in HER2-Enriched and Triple-Negative Breast Cancer. NPJ Precis. Oncol..

[95] Choi K., Hwang C., Gu S., Park M., Kim J., Park J., Ahn Y., Kim J., Song M., Song H. (2014). Cancer Cell Growth Inhibitory Effect of Bee Venom via Increase of Death Receptor 3 Expression and Inactivation of NF-Kappa B in NSCLC Cells. Toxins.

[96] de Bordon K.C.F., Cologna C.T., Fornari-Baldo E.C., Pinheiro-Júnior E.L., Cerni F.A., Amorim F.G., Anjolette F.A.P., Cordeiro F.A., Wiezel G.A., Cardoso I.A. (2020). From Animal Poisons and Venoms to Medicines: Achievements, Challenges and Perspectives in Drug Discovery. Front. Pharmacol..

[97] Robles-Loaiza A.A., Pinos-Tamayo E.A., Mendes B., Ortega-Pila J.A., Proaño-Bolaños C., Plisson F., Teixeira C., Gomes P., Almeida J.R. (2022). Traditional and Computational Screening of Non-Toxic Peptides and Approaches to Improving Selectivity. Pharmaceuticals.

[98] Cardoso M.H., Orozco R.Q., Rezende S.B., Rodrigues G., Oshiro K.G.N., Cândido E.S., Franco O.L. (2020). Computer-Aided Design of Antimicrobial Peptides: Are We Generating Effective Drug Candidates?. Front. Microbiol..

[99] Melo M.C.R., Maasch J.R.M.A., de la Fuente-Nunez C. (2021). Accelerating Antibiotic Discovery through Artificial Intelligence. Commun. Biol..

[100] Torres M.D.T., Cao J., Franco O.L., Lu T.K., de la Fuente-Nunez C. (2021). Synthetic Biology and Computer-Based Frameworks for Antimicrobial Peptide Discovery. ACS Nano.

